# G protein subunit alpha i2's pivotal role in angiogenesis

**DOI:** 10.7150/thno.92909

**Published:** 2024-03-03

**Authors:** Chao-wen Bai, Lu Lu, Jia-nan Zhang, Chengyu Zhou, Yi-chao Ni, Ke-ran Li, Jin Yao, Xiao-zhong Zhou, Chang-gong Lan, Cong Cao

**Affiliations:** 1 Department of Orthopedics, Clinical Research Center of Neurological Disease, The Second Affiliated Hospital of Soochow University, Institution of Neuroscience, Soochow University, Suzhou, China.; 2 Department of Joint Surgery and Geriatric Orthopedics, Affiliated Hospital of YouJiang Medical University for Nationalities, Guangxi Key Laboratory of Basic and Translational Research of Bone and Joint Degenerative Diseases, Guangxi Biomedical Materials Engineering Research Center for Bone and Joint Degenerative Diseases, Baise City, China.; 3 Department of Neuroscience, Case Western Reserve University, Cleveland, USA.; 4 The Fourth Medical School, Eye hospital, Nanjing Medical University, Nanjing, China.

## Abstract

Here we explored the potential role of Gαi2 (G protein subunit alpha i2) in endothelial cell function and angiogenesis.

**Methods:** Genetic methodologies such as shRNA, CRISPR/Cas9, dominant negative mutation, and overexpression were utilized to modify Gαi2 expression or regulate its function. Their effects on endothelial cell functions were assessed *in vitro*. *In vivo*, the endothelial-specific Gαi2 shRNA adeno-associated virus (AAV) was utilized to silence Gαi2 expression. The impact of this suppression on retinal angiogenesis in control mice and streptozotocin (STZ)-induced diabetic retinopathy (DR) mice was analyzed.

**Results:** Analysis of single-cell RNA sequencing data revealed *Gαi2* (*GNAI2*) was predominantly expressed in retinal endothelial cells and expression was increased in retinal endothelial cells following oxygen-induced retinopathy (OIR) in mice. Moreover, transcriptome analysis linking *Gαi2* to angiogenesis-related processes/pathways, supported by increased Gαi2 expression in experimental OIR mouse retinas, highlighted its possible role in angiogenesis. In various endothelial cell types, shRNA-induced silencing and CRISPR/Cas9-mediated knockout (KO) of Gαi2 resulted in substantial reductions in cell proliferation, migration, invasion, and capillary tube formation. Conversely, Gαi2 over-expression in endothelial cells induced pro-angiogenic activities, enhancing cell proliferation, migration, invasion, and capillary tube formation. Furthermore, our investigation revealed a crucial role of Gαi2 in NFAT (nuclear factor of activated T cells) activation, as evidenced by the down-regulation of NFAT-luciferase reporter activity and pro-angiogenesis NFAT-targeted genes (*Egr3*, *CXCR7*, and *RND1*) in Gαi2-silenced or -KO HUVECs, which were up-regulated in Gαi2-overexpressing endothelial cells. Expression of a dominant negative Gαi2 mutation (S48C) also down-regulated NFAT-targeted genes, slowing proliferation, migration, invasion, and capillary tube formation in HUVECs. Importantly, *in vivo* experiments revealed that endothelial Gαi2 knockdown inhibited retinal angiogenesis in mice, with a concomitant down-regulation of NFAT-targeted genes in mouse retinal tissue. In contrast, Gαi2 over-expression in endothelial cells enhanced retinal angiogenesis in mice. Single-cell RNA sequencing data confirmed increased levels of Gαi2 specifically in retinal endothelial cells of mice with streptozotocin (STZ)-induced diabetic retinopathy (DR). Importantly, endothelial Gαi2 silencing ameliorated retinal pathological angiogenesis in DR mice.

**Conclusion:** Our study highlights a critical role for Gαi2 in NFAT activation, endothelial cell activation and angiogenesis, offering valuable insights into potential therapeutic strategies for modulating these processes.

## Introduction

Angiogenesis is a fundamental and vital physiological process in the body 1, 2, serving as a critical mechanism to uphold the body's stability, and the functionality of cells, tissues, and organs 3-6. Vascular endothelial growth factor (VEGF) and other stimuli trigger the activation of existing vascular endothelial cells, often referred as “tip cells”. These specialized cells release proteases, facilitating the degradation of the basement membrane, thus enabling the detachment of endothelial cells from their original vessel walls 7. Tip cells exhibit slow proliferation and extend numerous filopodia, effectively guiding the direction of the angiogenic process 3-6. Detached endothelial cells subsequently proliferate and migrate into the surrounding matrix, coalescing to form sprouts that interconnect adjacent blood vessels 8. Endothelial cells trailing behind the tip cells continue their proliferation, contributing to the development of new capillary buds and the intricate network of fresh blood vessels 3-6. The intricate mechanisms governing the activation of endothelial cells and the process of angiogenesis remain largely unknown, serving as the primary research focus of our group 9-13.

Gαi proteins (G protein alpha i subunits), a subgroup of G protein family, are critical mediators of inhibitory signaling in the G protein-coupled receptor (GPCR) system 14-16. Upon activation by GPCR binding, Gαi proteins catalyze the exchange of GDP for GTP, leading to dissociation from the βγ subunits and subsequent downstream effects 14-16. Gαi proteins inhibit adenylyl cyclase (AC), resulting in decreased cyclic AMP (cAMP) levels, which impacts a wide range of cellular processes, including ion channel regulation and neurotransmitter release 14-16. Additionally, Gαi proteins modulate other signaling pathways, such as the MAPK (mitogen-activated protein kinase) and NFAT (nuclear factor of activated T cells) cascade, making them pivotal players in the regulation of diverse physiological processes and the focus of significant research and therapeutic interest 17. Three primary Gαi members are Gαi1, Gαi2 and Gαi3 18, 19.

Our group also established a pivotal role of Gαi proteins in mediating signaling by receptor tyrosine kinases (RTKs) and other non-GPCR receptors. Following epidermal growth factor (EGF) stimulation, Gαi1 and Gαi3 form a complex with activated EGFR to mediate downstream adaptor protein association and Akt-mammalian target of rapamycin (mTOR) cascade activation 20. Gαi1 and Gαi3 are also required for the signal transduction of other RTKs 10, 13, 21-23, including keratinocyte growth factor receptor (KGFR) 23, vascular endothelial growth factor receptor 2 (VEGFR2) 13, the BDNF receptor Tropomyosin receptor kinase B (TrkB) 22 and c-kit 10. Moreover, several non-RTK receptors also require Gαi1 and Gαi3 for downstream signal transduction, including Netrin-1 receptor CD146 (Cluster of Differentiation 146) 11, lipopolysaccharide (LPS) receptor Toll-like receptor 4 (TLR4)-and CD14 24 and IL-4 receptor IL-4R 25.

Recent investigations highlight the significant role of Gαi2 protein in the proliferation of cancer cells 26, 27. In a study by Chen *et al.,* Gαi2 over-expression was identified as a pivotal factor in promoting the growth of hepatocellular carcinoma (HCC) cells 27. Silencing or knockout (KO) of Gαi2 led to mitochondrial dysfunction, apoptosis, and growth arrest in HCC cells, impeding HCC xenograft growth in nude mice 27. Our recent study reported elevated *Gαi2* mRNA and protein expression in human gliomas, essential for *in vitro* and *in vivo* glioma cell growth 26. Mechanistically, Gαi2 enhanced the viability, proliferation, and mobility of human glioma cells, potentially through activation of the nuclear factor kappa B (NF-κB) pathway 26. Nonetheless, the specific involvement of Gαi2 in endothelial cell activation and angiogenesis, as well as the underlying mechanisms, have not been extensively studied to date.

## Methods

**Bioinformatics studies.** Analysis of publicly-available single-cell RNA sequencing (scRNA-seq) data from streptozotocin (STZ)-induced diabetic mice and oxygen-induced retinopathy (OIR) mice were conducted using data retrieved from the NCBI General Gene Expression Database (GEO) via GSE209872 and GSE150703. Initial processing of raw counts was performed using the R package Seurat (version 3.0), implementing quality control parameters such as minimum cells (min. cells = 3), minimum features (min. features = 100), and criteria for RNA features (nFeature_RNA >= 300, nFeature_RNA < 5000, mito.ratio < 0.20). Subsequently, identification of highly variable genes (top 5000) was executed via the FindVariableFeatures function. Principal component analysis (PCA) was applied to select the top 30 principal components for subsequent cluster analysis. Unsupervised hierarchical clustering, based on an appropriate resolution, was performed using the FindClusters function. Visualization of the clustered data was achieved through Uniform Manifold Approximation and Projection (UMAP) projections. Marker genes were identified, with additional markers of retinal cells retrieved from the GeneCards database to classify each cluster. Visualization of Gαi2 location and expression was accomplished using a density plot. Differential gene expression analysis was conducted to identify genes exhibiting up-regulated (***P*** value < 0.05 and avg_log2FC > 0.25) and down-regulated (***P*** value < 0.05 and avg_log2FC < -0.25) expression specifically in endothelial cells. Visualization of these findings was achieved using a volcano plot. Furthermore, publicly available transcriptome sequencing data for oxygen-induced retinopathy (OIR) mice were obtained from GEO using the search code GSE200195. Correlation analysis was conducted to determine genes most correlated with *Gαi2*. The top 100 correlated genes were subjected to enrichment analysis using The Database for Annotation, Visualization, and Integrated Discovery (DAVID). Pathways were ranked by ***P***-value, and the top 12 biological processes (BP) and KEGG (Kyoto Encyclopedia of Genes and Genomes) pathways were visualized using the ggplot2 package.

**Chemicals and reagents.** Polybrene, puromycin, ionomycin, cyclosporin A (CsA), and other chemicals were provided by Sigma-Aldrich (St. Louis, MO) unless otherwise specified. All antibodies were described in our previous studies 9-12, 26. Fluorescence dyes were provided by Thermo-Fisher Invitrogen (Soochow, China). All viral constructs and verified mRNA primers were supplied by Genechem (Shanghai, China).

**Cells.** The culture of various endothelial cell types in this study, including human umbilical vein endothelial cells (HUVECs), human microvascular endothelial cells (hRMECs), and human cerebral microvascular endothelial cells (hCMECs), was reported in our previous publications 9-12. Endothelial cells were cultured in DMEM/F-12 medium with high glucose and 10% FBS (Gibco, Suzhou, China), 5 μg/mL insulin, 5 μg/mL EGF, 5 ng/mL VEGF, and 24 μg/mL adenine, maintain a pro-angiogenic active state 12, and their genotype have been authenticated through short tandem repeat (STR) analysis, population doubling time assessments, and morphological examinations.

**Gαi2 small hairpin RNA (shRNA).** The lentiviral GV369 (Ubi-MCS-SV40-IRES-puromycin) construct (no tag) containing puromycin selection gene was reported in our previous studies 9, 12. It was utilized for shRNA experiment. Briefly, Gαi2 shRNA sequence was first inserted into the lentiviral construct. The latter, together with packaging vectors pHelper 1.0 and pHelper 2.0 (Genechem), were co-transduced to HEK-293 cells, thereby generating lentivirus. The virus, at MOI (multiplicity of infection) of 10, was added to cultured endothelial cells. Puromycin-containing complete medium was thereafter added to the cultured endothelial cells and stable cells were formed after 5-6 passages. Knockdown of Gαi2 in endothelial cells was verified at both mRNA and protein levels. Control cells were treated with scramble control scramble shRNA 26. In the context of *in vivo* studies, the mouse Gαi2 shRNA sequence was incorporated into an adeno-associated virus 5 (AAV5)-TIE1 construct 12, featuring the endothelial-specific promoter sequence of TIE1 (Genechem, Shanghai, China). Subsequently, the construct was transfected into HEK-293 cells to produce AAV, which was then administered to mice via intravitreal injection.

**Gαi2 knockout (KO).** The CRISPR-associated 9 (Cas9)-expressing stable HUVECs, reported in our previous studies 9, 12, were transduced with the previously-reported 26 lentivirus-packed CRISPR/Cas9-Gαi2-KO construct (containing puromycin selection gene). HUVECs were then selected by puromycin and distributed into 96-well plates. The stable cells were then subject to Gαi2 KO verification and thereafter single stable Gαi2 KO HUVECs (“koGαi2”) were formed. Control Cas9-expressing HUVECs were transduced with lentivirus-packed CRISPR/Cas9-KO construct containing scramble sgRNA sequence (“Cas9”). The same CRISPR/Cas9 procedures were employed to other endothelial cells (hRMECs and hCMEC/D3) to deplete Gαi2.

**Gαi2 over-expression and dominant negative mutation.** The lentivirus packed Gαi2-overexpressing GV369 construct was reported in our previous study 26 and was added to cultured endothelial cells. Puromycin-containing complete medium was thereafter added to the cultured endothelial cells, and stable cells formed after 5-6 passages. Gαi2 over-expression in the selected stable endothelial cells was verified at both mRNA and protein levels. Control cells were stably transduced with the empty GV369 vector (“Vec”). A site-directed mutagenesis (Genechem) using targeted primers was employed to generate the dominant negative S48C Gαi2 cDNA 28, and it was verified through DNA sequencing to confirm the presence of the desired mutation. The sequence was inserted into the GV369 construct, and the latter was stably transduced to endothelial cells. For in *in vivo* studies, the Gαi2 cDNA sequence was introduced into the AAV5-TIE1 construct. It was then transfected into HEK-293 cells to generate AAV, which was subsequently administered to mice via intravitreal injection.

**Cellular assays.** Cellular functional assays, including the assessment of cell proliferation through EdU (5-ethynyl-2'-deoxyuridine) staining of cell nuclei, “Transwell” *in vitro* cell migration and “Matrigel Transwell” *in vitro* cell invasion, *in vitro* capillary tube formation, nuclear TUNEL staining assay, JC-1 staining of mitochondrial depolarization the Caspase-3 and the Caspase-9 activity assays were described early 12, 13, 29-31. qRT-PCR (quantitative reverse transcription polymerase chain reaction) and Western blotting assays have been previously detailed in earlier publications 20, 32, 33. For "Transwell" and "Matrigel Transwell" assays, precisely 1.5 × 104 cells with specific genetic treatments were added into each chamber. In the capillary tube formation assay, 0.75 × 10^5^ cells per well were seeded onto pre-coated 24-well plates. For EdU, JC-1 and TUNEL staining assays, 5 × 10^4^ cells per well were seeded into 24-well plates. Crystal violet dye was utilized to stain the migrated/invaded cells. In cases where different proteins needed examination within the same set of lysates, parallel “sister” gels were employed. All the mRNA primers and viral constructs were sourced from Genechem (Shanghai, China). Figure **S1** included the uncropped blotting images of the study.

**NFAT-luciferase reporter assay.** HUVECs were transduced with a NFAT-luciferase reporter construct (Genechem), and stable cells were established after puromycin selection. Cells were then subjected to described treatments, and a luciferase assay was conducted, measuring both firefly luciferase (NFAT reporter) and Renilla luciferase (control) activities through the ONE-Glo™ Luciferase Assay System (Promega, Suzhou, China). The data was analyzed by normalizing firefly luciferase activity to Renilla luciferase activity.

**Animal models, virus injection in mouse retina and retinal staining.** The intravitreal injection of AAV involved adult C57BL/6 mice from the SLAC Laboratory Animal Center (Shanghai, China). These mice were housed in a clean specific pathogen-free (SPF) animal facility, maintained at a temperature of 24 ± 1°C, with a 12-hour light and dark cycle, and provided with free access to water and food. Anesthesia was induced as previously described 12, and a 33-gauge disposable needle was inserted through the sclera at the equator and posterior to the limbus, directly into the vitreous cavity. Approximately 0.1 μL of AAV was then injected into the vitreous cavity, targeting the needle above the optic nerve head. Twenty-one days after virus injection, the isolectin B4 (IB4) staining of retinal vasculature and NeuN (neuronal nuclear antigen) immunofluorescence staining of retinal ganglion cells (RGCs) were performed using the previously-described protocols 9-11. For the establishment of oxygen-induced retinopathy (OIR) mouse model, a previously-described method was utilized 13. Briefly, newborn C57BL/6 mice at P7 along with their nursing mothers were subjected to 75% oxygen exposure using a BioSpherix oxycycler (BioSpherix, Lacona, NY) for five days, followed by another five days of returning to room air. For the establishment of diabetic retinopathy (DR) animal model, C57B/6J mice, aged 4-5 weeks, underwent an overnight fasting period with unrestricted access to water. The subsequent day, the mice were administered intraperitoneally with freshly prepared streptozotocin (STZ) at a dosage of 60 mg/kg, dissolved in cold 100 mM pH 4.5 citrate buffer (Sigma-Aldrich), for five consecutive days. One week following the final STZ injection, tail vein blood glucose levels were evaluated. Mice displaying fasting blood glucose levels surpassing 300 mg/dL (16.6 mmol/L) were classified as diabetic and included in subsequent investigations. The "Mock" mice were administered citrate buffer injections. The assessment of retinal vascular leakage through Evans blue (EB) staining had also been previously outlined 12, 34. To isolate GFP-positive retinal endothelial cells from mouse retina, mice were sacrificed. Their eyes were carefully dissected to extract the retina. The tissues were then enzymatically digested via Collagenase I and Trypsin (Sigma) to obtain single-cell suspensions. Following digestion, cells were passed through a 70-µm Falcon cell strainer (Corning, Shanghai, China) and were centrifuged carefully. GFP-positive endothelial cells were then isolated using fluorescence-activated cell sorting (FACS) via BD FACSAria machine (BD, Shanghai, China). The isolated cells were then cultured into above mentioned medium. All animal protocols adhered to the guidelines established by the Institutional Animal Care and Use Committee and the Ethic Committee of Soochow University, in accordance with the provisions outlined in the ARVO (Association for Research in Vision and Ophthalmology) statement.

**Statistical analyses.** Statistical analyses were performed on normally-distributed data, presented as means ± standard deviation (SD). To assess significance, we employed one-way ANOVA followed by Scheffe's f-test for comparisons involving three or more groups, using SPSS 23.0, or the two-tailed unpaired t-test for comparisons between two groups, using Excel 2007. Statistical significance was defined as ***P***-values below 0.05.

## Results

### Gαi2 is highly expressed in endothelial cells and participates in angiogenesis and signaling

Among the models employed to investigate abnormal angiogenesis, oxygen-induced retinopathy (OIR) is a well-established experimental system 35, 36. The manipulation of oxygen levels in the OIR model induces a state of retinal ischemia followed by hyperoxia, resulting in the dysregulation of pro-angiogenic and anti-angiogenic factors. This imbalance triggers a cascade of molecular events, including the upregulation of vascular endothelial growth factor (VEGF), hypoxia-inducible factor (HIF), and various cytokines and growth factors, culminating in the formation of abnormal blood vessels in the retina 35, 36. Cellular responses such as endothelial cell proliferation, migration, and vascular remodeling contribute to the angiogenic process in OIR 35, 36.

**Figure 1:**
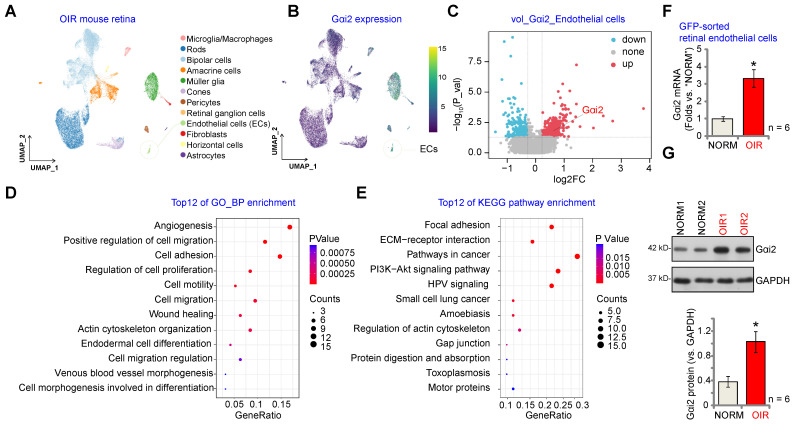
** Gαi2 is highly expressed in endothelial cells and participates in angiogenesis and signaling.** Visualization of the publicly available retinal scRNA-seq data (GEO: #GSE150703) of oxygen-induced retinopathy mice (OIR) and normoxic mice (NORM) using Uniform Manifold Approximation and Projection (UMAP) projections (**A**). The density plot illustrates the expression and spatial distribution of *Gαi2* in retinas, with the scale of expression density displayed on the right side of the plot (**B**). A differential gene volcano plot of endothelial cells compares differences between OIR and NORM group (**C**). Enrichment analysis shown possible biological process (BP, **D**) and KEGG pathways (**E**) of the top 100 co-expressing genes (CEGs) with Gαi2 in retinal tissues of OIR model mice. Expression of *Gαi2* mRNA (**F**) and protein (**G**) in GFP-sorted retinal cells of OIR mice and normoxic mice (NORM) mice was shown. Data were expressed as mean ± standard deviation (SD). Each group included six mice (n = 6, half male, half female) (**F** and **G**). *P < 0.05 versus “NORM” group.

We first conducted an analysis of publicly accessible single-cell RNA (scRNA) sequencing data (GEO: #GSE150703) comparing OIR mice and normoxic mice (NORM). The aim was to explore the cellular localization of *Gαi2* (*GNAI2*) within the retina. Through dimensionality reduction, clustering, and annotation techniques, we categorized individual cells from the entire retina into 12 distinct clusters (Figure **1A**). Subsequently, we utilized Uniform Manifold Approximation and Projection (UMAP) projections to visualize these clusters (Figure **1A**). As depicted in Figure **1B**, *Gαi2* is ubiquitously expressed across all cell types within the mouse retina, with significantly elevated levels observed within endothelial cells. This suggests a potentially functional role for *Gαi2* in endothelial cells. Furthermore, *Gαi2* expression in pericytes and Müller glia is also relatively high compared to other cell types (Figure **1B**). A volcano plot depicting the differential gene expression was generated comparing OIR and NORM mice groups that revealed a significant up-regulation of *Gαi2* (Figure **1C**) specifically within retinal endothelial cells of the OIR group.

Next, we performed correlation analysis using publicly accessible transcriptome sequencing data (GEO: #GSE200195) from retinal tissue of OIR model mice to uncover genes co-expressed with *Gαi2*. The top 100 co-expressing genes (CEGs) exhibiting the closest correlation were subjected to enrichment analysis, revealing the top 12 potential biological processes (BPs) in which *Gαi2* might be involved. The analysis highlighted that *Gαi2* CEGs were enriched in key biological processes for angiogenesis, including "Angiogenesis" "Cell migration", "Cell adhesion" and "Cell proliferation" (Figure **1D**). The Kyoto Encyclopedia of Genes and Genomes (KEGG) analyses conducted on *Gαi2* CEGs also revealed the top 12 pathways (Figure **1E**). Among these pathways, several are recognized for their significance in endothelial cell activation and angiogenesis, including "Focal adhesion", "ECM-receptor interaction" and "Pathways in cancer" (Figure **1E**).

To validate the above bioinformatics results, a previously-described OIR mouse model was utilized 13. P2 mice were anesthetized and a 33-gauge needle was inserted through the sclera into the vitreous cavity, where 0.1 μL of AAV5-TIE1-GFP was injected above the optic nerve head 12. P7 mice were then subjected to 75% oxygen exposure for five days, followed by five days at room air. At P17, OIR or mock control mice were sacrificed to extract retinas, which were enzymatically digested and passed through a 70-µm cell strainer before GFP-positive endothelial cells were sorted using FACS. These cells were then cultured. As demonstrated, *Gαi2* mRNA (Figure **1F**) and protein (Figure **1G**) expression levels were notably elevated in GFP-positive endothelial cells of OIR mice compared to those from the mock control mice.

### Gαi2 silencing impedes *in vitro* angiogenesis in cultured endothelial cells

**Figure 2:**
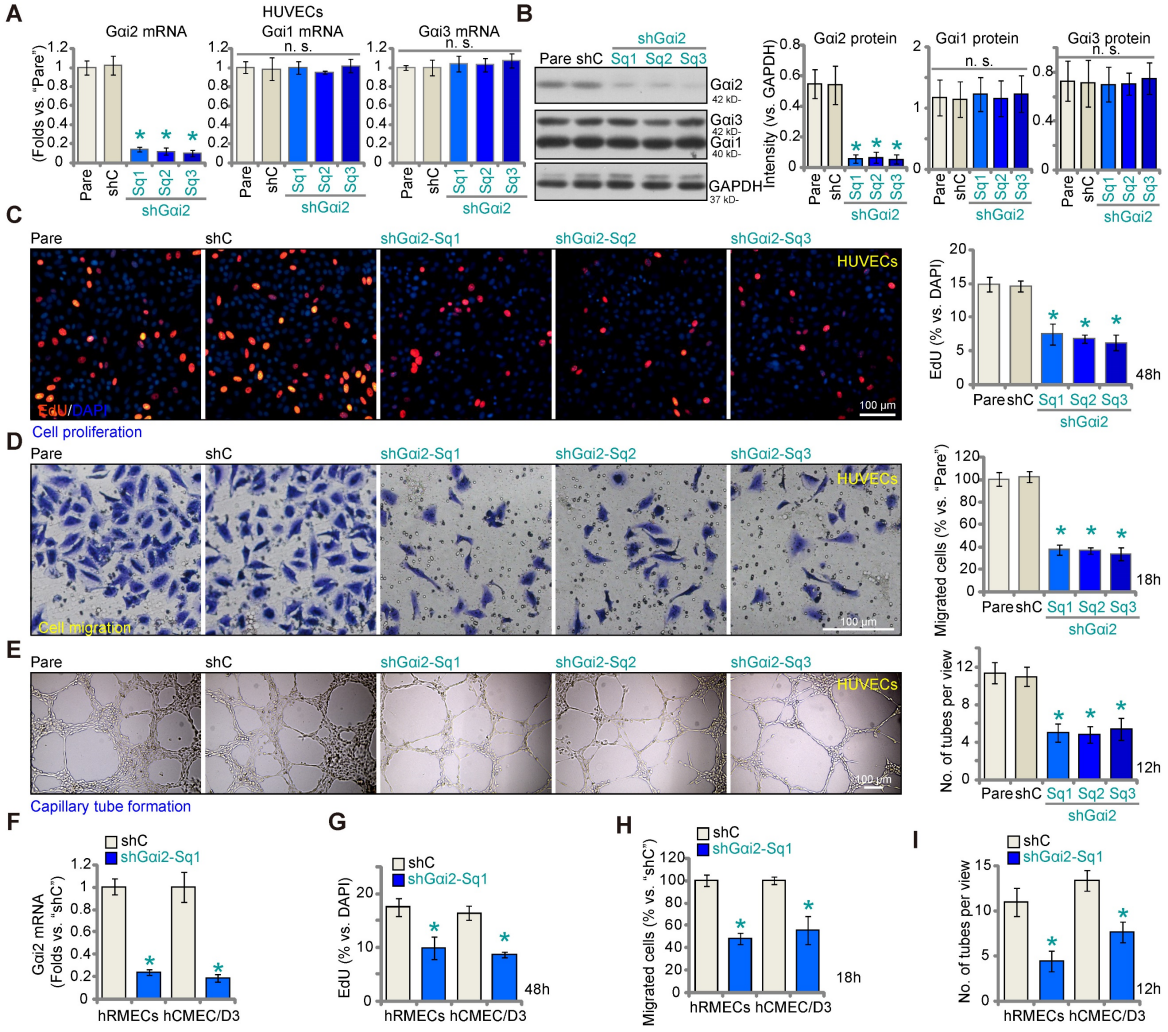
** Gαi2 silencing impedes *in vitro* angiogenesis in cultured endothelial cells.** The mRNA (**A**) and protein (**B**) expression of Gαi1/2/3 in stable human umbilical vein endothelial cells (HUVEC) with the listed Gαi2 shRNA (shGαi2-Sq1, shGαi2-Sq2 or shGαi2-Sq3), scramble shRNA (“shC”) or in parental control (“Pare”) HUVECs were shown; The exact same number of the above HUVECs were maintained under complete medium and cultivated for designated hours, *in vitro* cell proliferation (by measuring nuclear EdU incorporation, **C**), migration (“Transwell” assays, **D**) and capillary tube formation (**E**) were examined. *Gαi2* mRNA expression in human retinal microvascular endothelial cells (hRMECs) and human cerebral microvascular endothelial cells (hCMEC/D3) with shC or shGαi2-Sq1 was shown (**F**); The endothelial cells were maintained under complete medium and cultivated for designated hours, *in vitro* cell proliferation (**G**), migration (**H**) and capillary tube formation (**I**) were tested similarly, with results quantified. Data were expressed as mean ± standard deviation (SD). The quantifications were from five biological repeats (n = 5). *P < 0.05 versus “shC” group. “n.s.” stands for non-statistical differences (***P*** > 0.05). Scale bar = 100 μm.

Aiming to understand the potential role of Gαi2 in angiogenesis, we first employed a lentiviral shRNA method to silence Gαi2. A set of three different lentivirus-packed shRNAs, shGαi2-Sq1, shGαi2-Sq2 and shGαi2-Sq3 (with non-overlapping sequences), were separately transduced into cultured HUVECs, and stable cells formed after puromycin-based selection. As compared to the parental control (“Pare”) HUVECs and scramble shRNA (“shC”)-expressing HUVECs, *Gαi2* mRNA (Figure **2A**) and protein (Figure **2B**) expression was substantially decreased in stable HUVECs with the described Gαi2 shRNAs. The mRNA and protein expression of two other members of the Gαi family, Gαi1 and Gαi3, was however not significantly altered in shGαi2 HUVECs (Figure **2A** and **B**). Importantly, Gαi2 shRNA impeded HUVEC proliferation *in vitro* and decreased the percentage of EdU-positive nuclei (Figure **2C**). “Transwell” assay results demonstrated that *in vitro* migration (Figure **2D**) was slowed after Gαi2 silencing in HUVECs. Moreover, capillary tube formation was inhibited after shRNA-induced knockdown of Gαi2 (Figure **2E**). These results demonstrate that Gαi2 silencing impedes *in vitro* angiogenesis in HUVECs. The shC control failed to alter proliferation (Figure 2**C**) migration (Figure **2D**) and capillary tube formation (Figure **2E**) in HUVECs.

To silence Gαi2 in other endothelial cell types, including human retinal microvascular endothelial cells (hRMECs) and human cerebral microvascular endothelial cells (hCMEC/D3) 12, the lentivirus-packed shGαi2-Sq1 was transduced and stable cells established following puromycin selection. As compared to the endothelial cells with shC, *Gαi2* mRNA expression was dramatically decreased in shGαi2-Sq1-expressing hRMECs and hCMEC/D3 (Figure **2F**). Importantly, *in vitro* cell proliferation (nuclear EdU incorporation, Figure **2G**), migration (Figure **2H**) and capillary tube formation (Figure **2I**) were inhibited following Gαi2 silencing in hRMECs and hCMEC/D3. These results further support the pro-angiogenic activity Gαi2 *in vitro*.

### Gαi2 silencing provokes apoptosis in cultured endothelial cells

**Figure 3:**
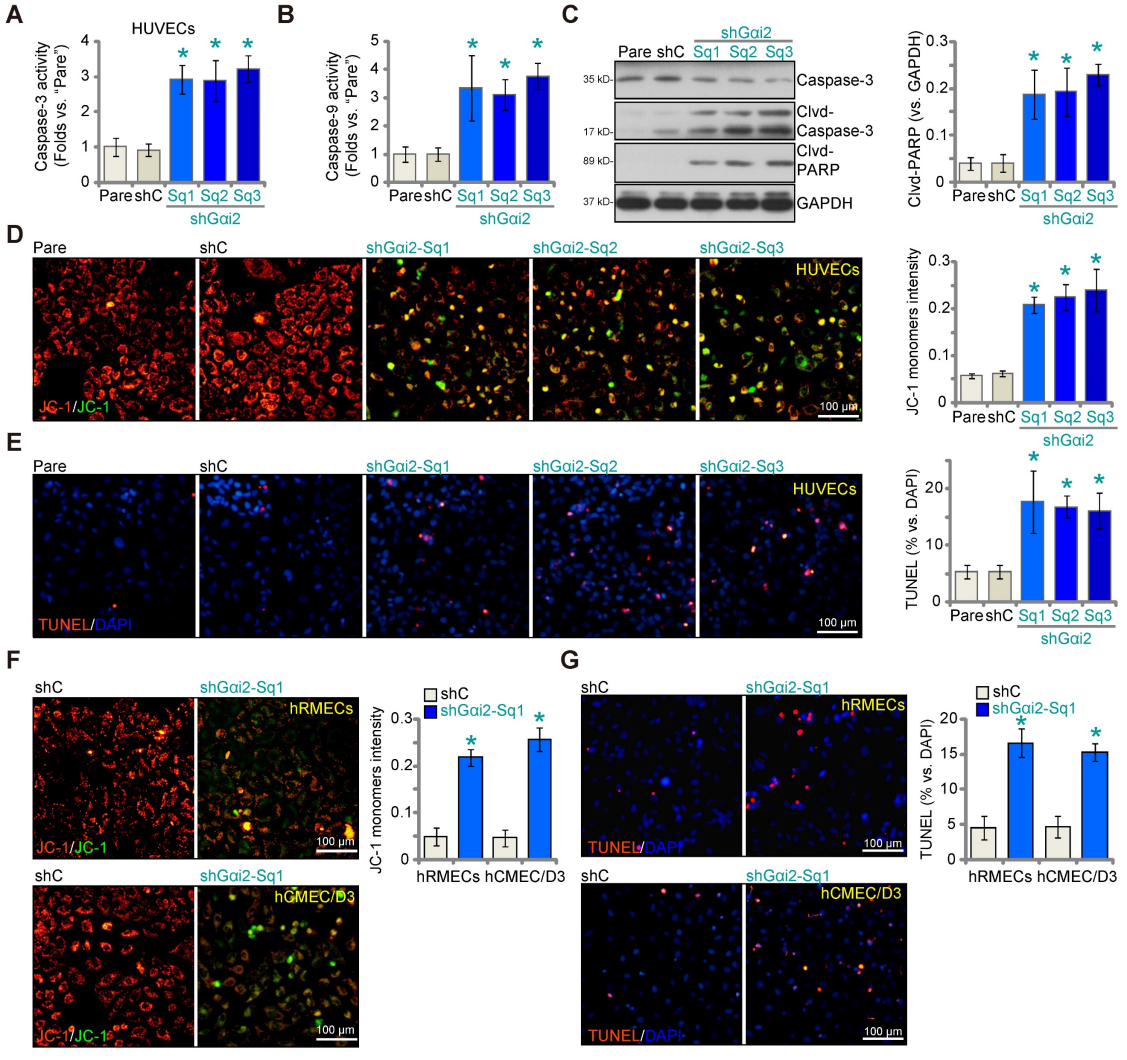
** Gαi2 silencing provokes apoptosis in cultured endothelial cells.** The exact same number of HUVECs with the listed Gαi2 shRNA (shGαi2-Sq1, shGαi2-Sq2 or shGαi2-Sq3), scramble shRNA (“shC”) or the parental control (“Pare”) HUVECs were cultivated for 60h, Caspase-3/9-PARP activation/cleavages were tested (**A**-**C**); The depolarization of mitochondria was examined by quantifying aggregation of JC-1 green monomers (**D**); Cell apoptosis was tested by measuring TUNEL-positive nuclei ratio (**E**). The exact same number of hRMECs or hCMEC/D3 with shC or shGαi2-Sq1 were cultivated for 60h, depolarization of mitochondria and apoptosis were respectively examined via JC-1 staining (**F**) and nuclear TUNEL staining assays (**G**). Data were expressed as mean ± standard deviation (SD). Quantifications were from five biological repeats (n = 5). *P < 0.05 versus “shC” group. Scale bar = 100 μm.

We next examined whether Gαi2 silencing would provoke apoptosis in endothelial cells. As shown in cultured HUVECs, Gαi2 silencing by lentiviral shGαi2-Sq1, shGαi2-Sq2 or shGαi2-Sq3 (see Figure **1**), increased Caspase-3 (Figure **3A**) and Caspase-9 activity (Figure **3B**). Additionally, we observed enhanced cleavage of Caspase-3 and Poly(ADP-ribose) polymerase 1 (PARP) in shGαi2 HUVECs (Figure **3C**). Silencing of Gαi2 in HUVECs also led to mitochondrial depolarization, as evidenced by the aggregation of JC-1 green monomers (Figure **3D**), a characteristic indicator of mitochondrial apoptotic pathway activation 37. Gαi2 silencing induced a moderate yet significant level of apoptosis in cultured HUVECs, as indicated by an approximately threefold increase in the percentage of TUNEL-positive nuclei in cells expressing shGαi2-Sq1, shGαi2-Sq2, or shGαi2-Sq3 (Figure **3E**). In other endothelial cell types, hRMECs and hCMEC/D3, Gαi2 silencing using shGαi2-Sq1 (see Figure **1**) similarly induced mitochondrial depolarization (Figure **3F**) and apoptosis, as assessed by the increased ratio of TUNEL-positive nuclei (Figure **3G**).

### Complete Gαi2 knockout (KO) leads to significant anti-angiogenic activity in cultured endothelial cells

The results presented above demonstrate that silencing Gαi2 using shRNA inhibits *in vitro* angiogenesis in cultured endothelial cells. To rule out any potential off-target effects of the shRNAs and to achieve complete Gαi2 depletion, we employed the CRISPR/Cas9 strategy. Specifically, Cas9-expressing HUVECs, as described in our earlier publications 9-11, were transduced with a lentivirus-packed CRISPR/Cas9-Gαi2-KO construct 26, which included a sequence of sgRNA targeting human *Gαi2*. Subsequently, we established single stable Gαi2 KO HUVECs, referred to as "koGαi2," following puromycin selection and verification of Gαi2 KO. As a control treatment, Cas9-expressing HUVECs were transduced with a lentivirus-packed CRISPR/Cas9-KO construct containing a scramble sgRNA sequence, denoted as "Cas9."

**Figure 4:**
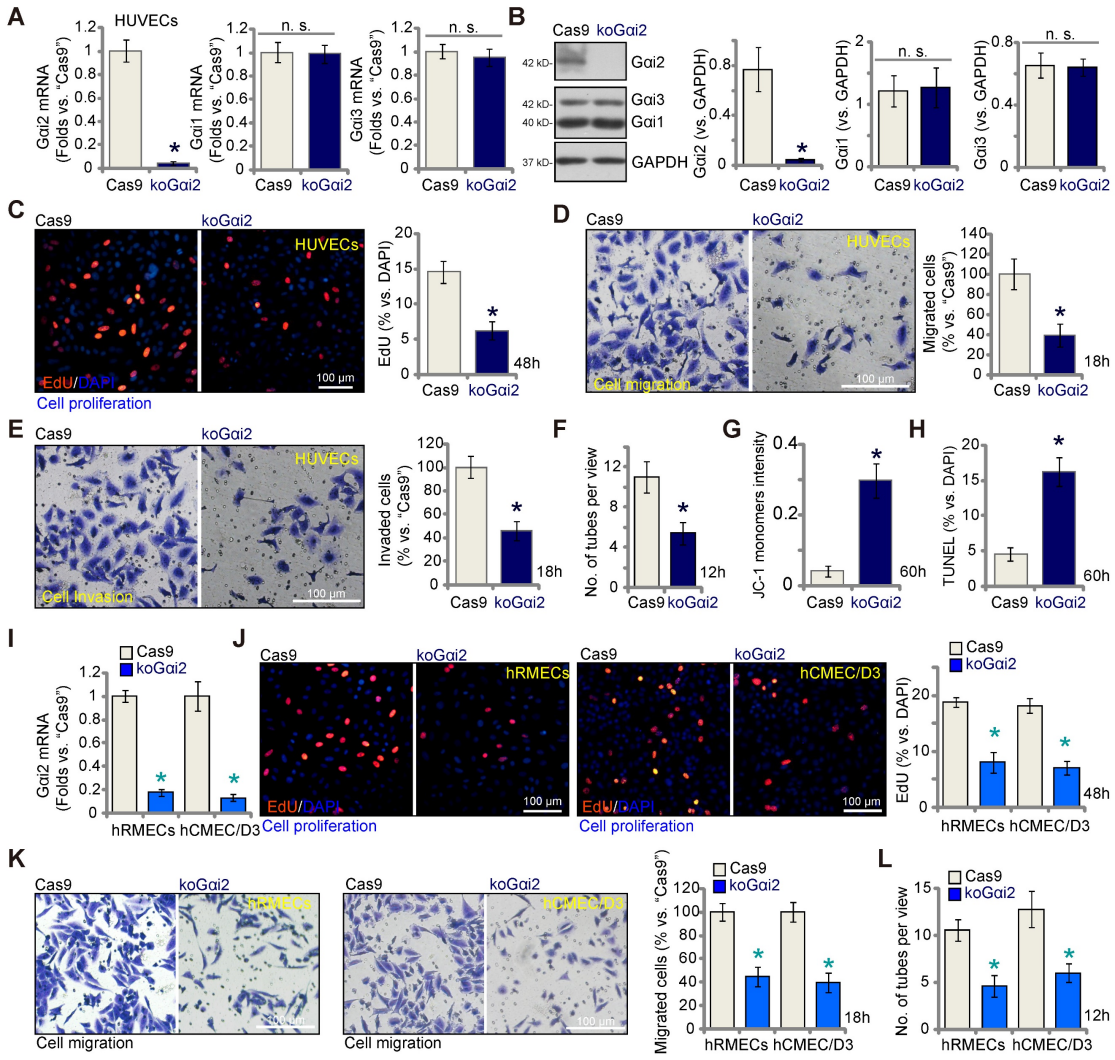
** Complete Gαi2 knockout (KO) leads to significant anti-angiogenic activity in cultured endothelial cells.** The mRNA (**A**) and protein (**B**) expression of Gαi1/2/3 in single stable HUVECs with both Cas9-expressing construct plus the CRISPR/Cas9-Gαi2-KO construct (“koGαi2”) or the control construct (“Cas9”) was shown. The exact same number of the above HUVECs were cultivated for designated hours, *in vitro* cell proliferation (by measuring nuclear EdU incorporation, **C**), migration (“Transwell” assays, **D**), invasion (“Matrigel Transwell” assays, **E**) and capillary tube formation (**F**) were tested; Depolarization of mitochondria and apoptosis were respectively examined via measuring JC-1 monomer intensity (**G**) and nuclear TUNEL ratio (**H**). *Gαi2* mRNA expression in hRMECs or hCMEC/D3 with same CRISPR/Cas9 genetic treatment, “koGαi2” or “Cas9”, was shown (**I**); The exact same number of cells were cultivated for designated hours, *in vitro* cell proliferation (**J**), migration (**K**) and capillary tube formation (**L**) were tested similarly. Data were expressed as mean ± standard deviation (SD). Quantifications were from five biological repeats (n = 5). *P < 0.05 versus “Cas9” group. “n.s.” stands for non-statistical differences (***P*** > 0.05). Scale bar = 100 μm.

*Gαi2* mRNA expression, as well as protein expression was effectively depleted in koGαi2 HUVECs (Figure **4A** and **B**), while Gαi1 and Gαi3 expression remained unaffected (Figure **4A** and **B**). Gαi2 KO in HUVECs induced a strong anti-angiogenic effect, significantly inhibiting *in vitro* cell proliferation (as indicated by nuclear EdU incorporation, Figure **4C**), migration (Figure **4D**), invasion (Figure **4E**), and capillary tube formation (Figure **4F**). Furthermore, Gαi2 KO led to mitochondrial depolarization and the aggregation of JC-1 green monomers in HUVECs (Figure **4G**). Increased TUNEL-nuclei staining provided further evidence of apoptosis activation in Gαi2 KO HUVECs (Figure **4H**). In both hRMECs and hCMEC/D3, Gαi2 KO was achieved using the same CRISPR/Cas9 strategy (Figure **4I**), resulting in impaired *in vitro* angiogenesis and reduced cell proliferation (nuclear EdU incorporation, Figure **4J**), migration (Figure **4K**), and capillary tube formation (Figure **4L**).

### Gαi2 over-expression promotes angiogenesis in cultured endothelial cells

**Figure 5:**
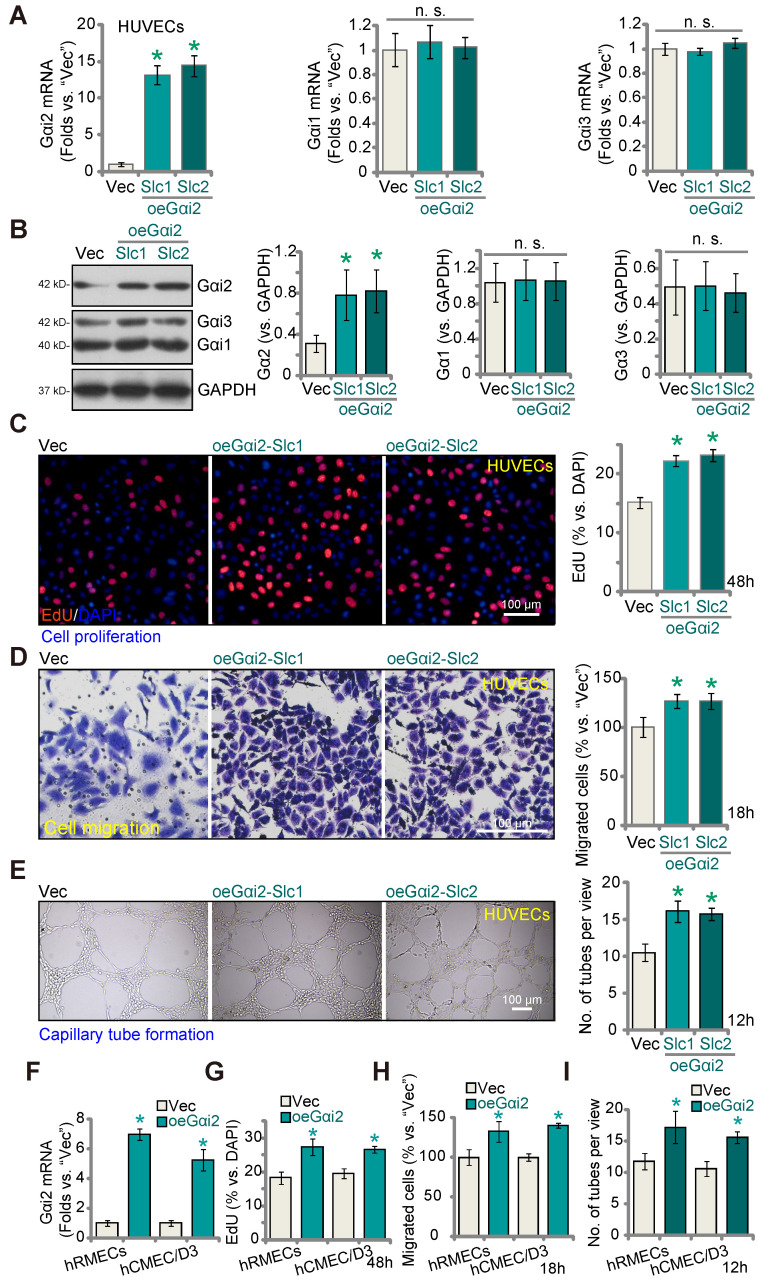
** Gαi2 over-expression promotes angiogenesis in cultured endothelial cells.** The mRNA (**A**) and protein (**B**) expression of Gαi1/2/3 in HUVECs with the Gαi2-overexpressing construct (oeGαi2-Slc1 and oeGαi2-Slc2, representing two stable selections) or empty vector (“Vec”) was shown; The exact same number of the above HUVECs were cultivated for designated hours, *in vitro* cell proliferation (by measuring nuclear EdU incorporation, **C**), migration (“Transwell” assays, **D**), and capillary tube formation (**E**) were tested. *Gαi2* mRNA expression in hRMECs or hCMEC/D3 with the Gαi2-overexpressing construct (oeGαi2) or empty vector (“Vec”) was shown (**F**); The exact same number of cells were cultivated for designated hours, *in vitro* cell proliferation (**G**), cell migration (**H**) and capillary tube formation (**I**) were tested similarly. Data were expressed as mean ± standard deviation (SD). Quantifications were from five biological repeats (n = 5). *P < 0.05 versus “Vec” group. “n.s.” stands for non-statistical differences (***P*** > 0.05). Scale bar = 100 μm.

Given that silencing or KO of Gαi2 results in anti-angiogenic activity, we hypothesized that elevating Gαi2 expression could enhance angiogenesis in cultured endothelial cells. To investigate this hypothesis, we transduced HUVECs with a lentivirus-packed Gαi2-overexpressing construct known as "oeGαi2" 26, and two stable cell lines, oeGαi2-Slc1 and oeGαi2-Slc2, were established through selection. In comparison to cells transfected with the vector control ("Vec"), the oeGαi2-expressing HUVECs exhibited a remarkable increase in *Gαi2* mRNA levels, exceeding 10- to 15-fold elevation (Figure **5A**), while *Gαi1* and *Gαi3* mRNA levels remained unchanged (Figure **5A**). Notably, Gαi2 protein upregulation was also observed in oeGαi2-Slc1 and oeGαi2-Slc2 HUVECs, with Gαi1 and Gαi3 protein expression remaining unaltered (Figure **5B**). Ectopic over-expression of Gαi2 induced pro-angiogenic activity, as evidenced by a significant increase in cell proliferation (tested via the ratio of EdU-positive nuclei) in oeGαi2-Slc1 and oeGαi2-Slc2 HUVECs (Figure **5C**). Furthermore, oeGαi2 treatment accelerated *in vitro* migration (Figure **5D**) and promoted capillary tube formation (Figure **5E**) in HUVECs.

In other endothelial cell types, hRMECs and hCMEC/D3, stable over-expression of Gαi2 using the same lentiviral construct, oeGαi2, similarly resulted in a substantial upregulation of *Gαi2* mRNA (Figure **5F**). With Gαi2 over-expression, endothelial cells exhibited increased cell proliferation (nuclear EdU incorporation, Figure **5G**), accelerated cell migration (Figure **5H**), and augmented capillary tube formation (Figure **5I**).

### Gαi2 is important for activation of the transcription factor NFAT in endothelial cells

**Figure 6:**
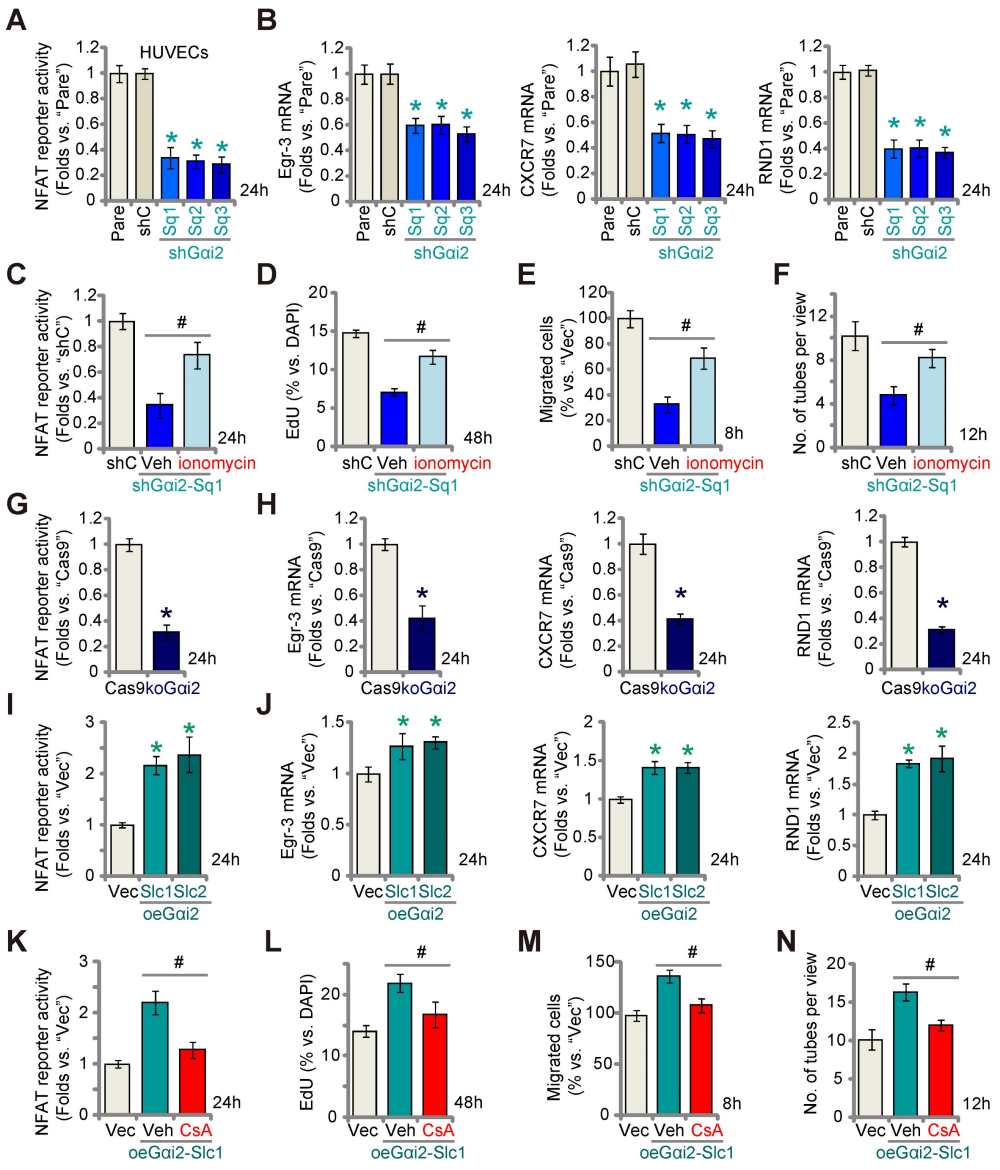
** Gαi2 is important for activation of the transcription factor NFAT in endothelial cells.** Stable HUVECs with the listed Gαi2 shRNA (shGαi2-Sq1, shGαi2-Sq2 or shGαi2-Sq3), scramble nonsense shRNA (“shC”), Cas9-expressing construct plus the CRISPR/Cas9-Gαi2-KO construct (“koGαi2”), the control construct (“Cas9”), the Gαi2-overexpressing construct (oeGαi2-Slc1 and oeGαi2-Slc2), empty vector (“Vec”) were established, the NFAT-luciferase reporter activity was tested (**A**, **G** and **I**), and expression of listed mRNAs tested as well (**B**, **H** and **J**). Stable shGαi2-Sq1-expressing HUVECs were treated with ionomycin (5 µM) or vehicle control (DMSO) for listed hours, the NFAT-luciferase reporter activity was tested (**C**); The *in vitro* cell proliferation (by measuring nuclear EdU incorporation, **D**), migration (“Transwell” assays, **E**) and capillary tube formation (**F**) were measured, with results quantified. Stable oeGαi2-Slc1-expressing HUVECs were treated with cyclosporin A (CsA, 2.5 µM) or vehicle control (DMSO) for listed hours, the NFAT-luciferase reporter activity was tested (**K**); The *in vitro* cell proliferation (**L**), cell migration (**M**) and capillary tube formation (**N**) were tested similarly, with results quantified. “Pare” stands for the parental control cells. Data were expressed as mean ± standard deviation (SD). Quantifications were from five biological repeats (n = 5). *P < 0.05 versus “shC”/”Cas9”/”Vec” control cells (**A**, **B**, **G**-**J**). ^#^ ***P*** < 0.05 versus “Veh” treatment (**C**-**F**, **K**-**N**).

A previous study demonstrated the significance of Gαi2 in triggering the activation of the transcription factor NFAT (nuclear factor of activated T cells) within skeletal muscle cells 38. NFAT signaling plays a pivotal role in endothelial cell activation and angiogenesis 39, 40. Therefore, we set out to study whether Gαi2 has an impact on NFAT activation in endothelial cells. As shown, Gαi2 silencing by targeted shRNAs, shGαi2-Sq1, shGαi2-Sq2 and shGαi2-Sq3, potently decreased the NFAT-luciferase reporter activity in cultured HUVECs (Figure **6A**). Expression of NFAT-dependent and pro-angiogenic genes, including *Egr3* (*early growth response 3*), *CXCR7* (*C-X-C chemokine receptor 7*) and RND1 (*Rho family GTPase 1*) 40, 41, was also down-regulated by the shRNAs (Figure **6B**). Ionomycin, known for its ability to activate the calcineurin-NFAT cascade 42, reinstated NFAT reporter activity in Gαi2-silenced HUVECs (shGαi2-Sq1) (Figure **6C**). Additionally, ionomycin significantly mitigated the inhibitory effects of Gαi2 shRNA on HUVECs proliferation (Figure **6D**), migration (Figure **6E**), and tube formation (Figure **6F**).

Similarly, CRISPR/Cas9 KO of Gαi2 largely decreased NFAT-luciferase reporter activity (Figure **6G**) and down-regulated *Egr3*, *CXCR7* and *RND1* (Figure **6H**) in cultured HUVECs. In contrast, in the Gαi2-overexpressing HUVECs, oeGαi2-Slc1 and oeGαi2-Slc2, NFAT-luciferase reporter activity was strengthened (Figure **6I**) and expression of NFAT-dependent genes was up-regulated (Figure **6J**). CsA, an established NFAT inhibitor 43, 44, blocked the increased NFAT reporter activity observed in oeGαi2-Slc1 HUVECs (Figure **6K**). Functionally, the Gαi2 over-expression-induced enhancement in HUVECs proliferation (Figure **6L**), migration (Figure **6M**), and capillary tube formation (Figure **6N**) were reversed by CsA. These results further support that Gαi2 promotes endothelial cell activation through the upregulation of NFAT signaling.

### A dominant negative Gαi2 hinders *in vitro* angiogenesis in cultured endothelial cells

**Figure 7:**
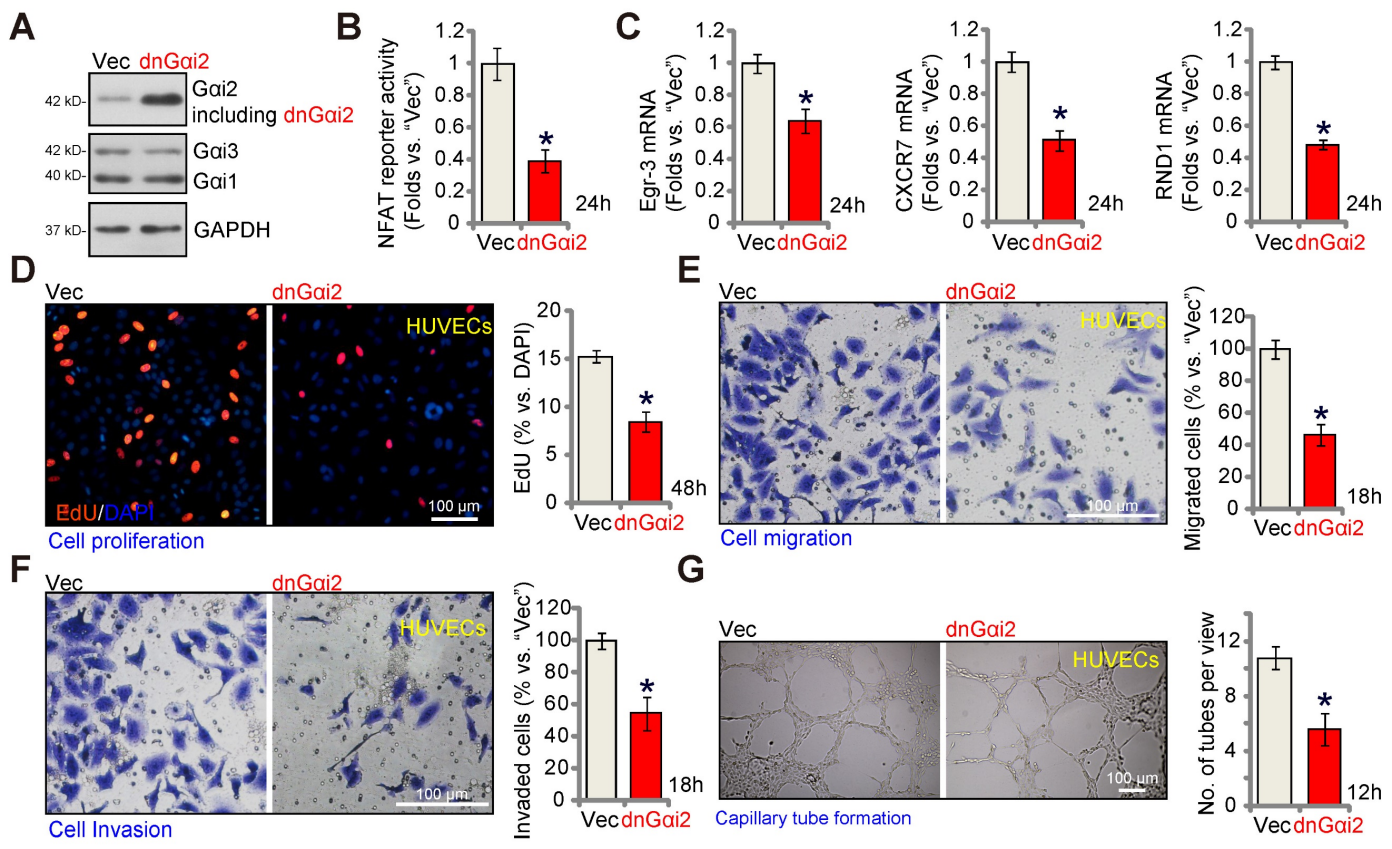
** A dominant negative Gαi2 hinders *in vitro* angiogenesis in cultured endothelial cells.** The expression of Gαi1/2/3 in stable HUVECs with the dominant negative mutant Gαi2 (S48C, “dnGαi2”) or the empty vector (“Vec”) was shown (**A**); The NFAT-luciferase reporter activity (**B**) and expression of listed mRNAs (**C**) were measured as well. The exact same number of the above HUVECs were cultivated for designated hours, *in vitro* cell proliferation (by measuring nuclear EdU incorporation, **D**), migration (“Transwell” assays, **E**), invasion (“Matrigel Transwell” assays, **F**) and capillary tube formation (**G**) were also tested, with results quantified. Data were expressed as mean ± standard deviation (SD). Quantifications were from five biological repeats (n = 5). *P < 0.05 versus “Vec” control cells.

The key step for the activation of heterotrimeric G proteins involves the GTP binding process, causing the separation of subunits and producing GTP-bound α subunits alongside liberated βγ complexes. A dominant negative mutation, Gαi2 (S48C), is known to completely disrupt GTP binding and inhibit Gαi2 activation 28, 45. A lentiviral dominant negative Gαi2 (dnGαi2) construct was stably transduced into HUVECs, and stable cells formed after puromycin selection. Western blot assay results, Figure **7A,** confirmed expression of dnGαi2 alongside the wild type Gαi2 in the stable HUVECs, and Gαi1 and Gαi3 expression remained unchanged (Figure **7A**). Importantly, dnGαi2 decreased NFAT-luciferase reporter activity (Figure **7B**) and down-regulated NFAT-dependent gene expression (*Egr3*, *CXCR7* and *RND1*) (Figure **7C**). The dnGαi2 also induced anti-angiogenic activity HUVECs, inhibiting proliferation (EdU ratio reduction, Figure **7D**), *in vitro* cell migration (Figure **7E**) and invasion (Figure **7F**). Moreover, capillary tube formation was also inhibited in dnGαi2-expressing HUVECs (Figure **7G**).

### Endothelial knockdown of Gαi2 inhibits retinal angiogenesis in mice

**Figure 8:**
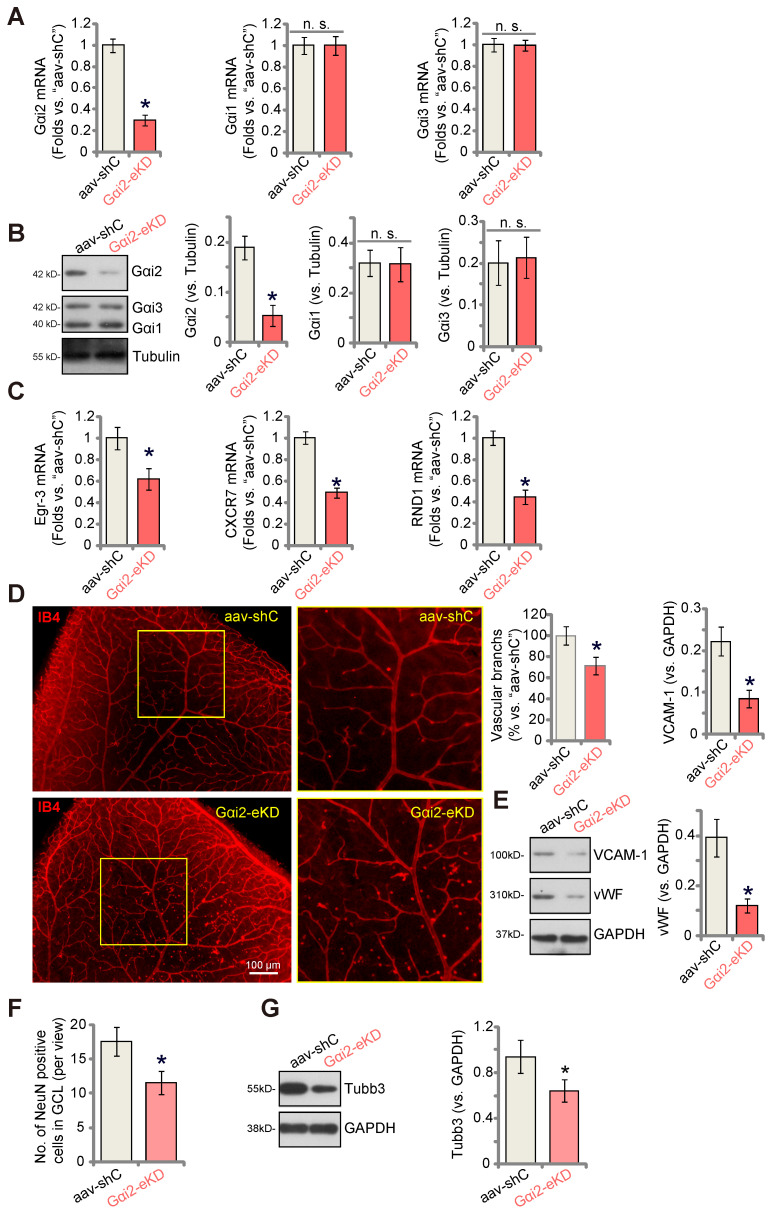
** Endothelial knockdown of Gαi2 inhibits retinal angiogenesis in mice.** Adult C57BL/6 mice (32.40 ± 2.32-day old, three male two female in each group) received intravitreal injections of either murine AAV5-TIE1-Gαi2 shRNA (“Gαi2-eKD”, 0.1 μL) or AAV5-TIE1 scramble control shRNA (“aav-shC”, 0.1 μL). After a twenty-one-day period, expression of listed mRNAs and proteins in murine retinal tissues was tested (**A**-**C**, **E** and **G**). Additionally, the retinal vasculature was visualized using retinal isolectin B4 (IB4) staining (**D**). In the retinal slides, NeuN/DAPI immunofluorescence staining was performed, and the quantification of NeuN-positive RGCs within GCL was conducted (**F**). “GCL”: ganglion cell layer, “ONL”: Outer nuclear layer, “INL”: Inner nuclear layer. Data were expressed as mean ± standard deviation (SD). Quantifications were from five biological repeats (n = 5). *P < 0.05 versus “aav-shC” group. Scale bar = 50/100 μm.

To explore the potential influence of Gαi2 on *in vivo* angiogenesis, we carried out experiments using the mouse retinal vasculature, as previously described 46. Initially, adult mice underwent intravitreal injection of murine AAV5-TIE1-Gαi2 shRNA (no Taq), which was driven by the endothelial cell-specific TIE1 promoter 9, 12. This intervention effectively reduced Gαi2 expression exclusively in endothelial cells, referred to as “Gαi2-eKD”. As a genetic control treatment, murine AAV5-TIE1-scramble control shRNA (“aav-shC”) 46 was administered to the mouse retina. Three weeks after the virus injection, we collected murine retinal tissues and analyzed tissue lysates. In Gαi2-eKD mice, *Gαi2* mRNA (Figure **8A**) and protein (Figure **8B**) levels were significantly decreased, while Gαi1 and Gαi3 mRNA (Figure **8A**) and protein (Figure **8B**) levels remained unchanged. Additionally, endothelial knockdown of Gαi2 led to a reduction in the expression of NFAT-dependent genes (*Egr3*, *CXCR7* and *RND1*) in the retinal tissues (Figure **8C**).

Examination of retinal vasculature through IB4 staining demonstrated a strong inhibition of angiogenesis in the mouse retina following endothelial knockdown of Gαi2 (Figure **8D**). Gαi2-eKD mice displayed a marked decrease in the number of retinal vascular branches and branch points (Figure **8D**). Furthermore, the endothelial marker proteins, von Willebrand factor (vWF) and VCAM-1, exhibited reduced expression in retinal tissues following Gαi2-eKD (Figure **8E**). Therefore, endothelial knockdown of Gαi2 effectively suppressed retinal angiogenesis in mice.

When retinal angiogenesis is disrupted, retinal ganglion cells (RGCs), the neuronal cells within the retina, become highly susceptible to cell death due to the deprivation of oxygen, energy, and essential nutrients 9, 12. This constitutes a fundamental pathology in various retinal disorders, including diabetic retinopathy (DR), retinopathy of prematurity (ROP), and neovascular glaucoma 47. We thus conducted an analysis to test whether the endothelial knockdown of Gαi2 had an impact on RGCs within the mouse retina. Quantification of retinal immuno-fluorescence images revealed a reduction in the number of NeuN-positive RGCs within the ganglion cell layer (GCL) following endothelial Gαi2 knockdown (Figure **8F**). Tubb3 (beta-III tubulin) is a marker for the structural integrity and health of RGCs in the retina. We showed that the expression of Tubb3 protein was down-regulated in the retinal tissues of Gαi2-eKD mice (Figure **8G**), further supporting RGC degeneration.

### Endothelial over-expression of Gαi2 strengthens retinal angiogenesis in mice

**Figure 9:**
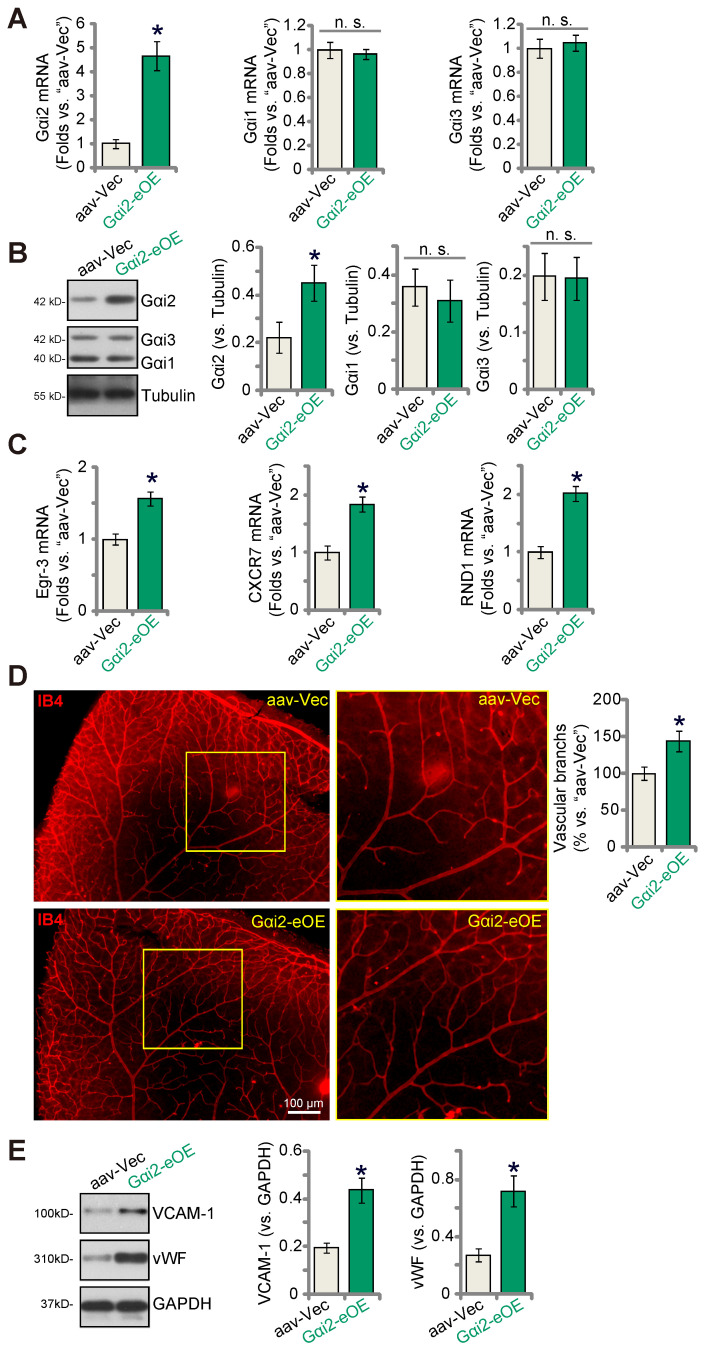
** Endothelial over-expression of Gαi2 strengthens retinal angiogenesis in mice.** Adult C57BL/6 mice (31.30 ± 2.45 day old, three male two female in each group) received intravitreal injections of either murine AAV5-TIE1-Gαi2-expressing construct (“Gαi2-eOE”, 0.1 μL) or AAV5-TIE1-vector (“aav-Vec”, 0.1 μL). After a twenty-one-day period, expression of listed mRNAs and proteins in murine retinal tissues was tested (**A**-**C**, and **E**). Additionally, the retinal vasculature was visualized using retinal isolectin B4 (IB4) staining (**D**). Data were expressed as mean ± standard deviation (SD). Quantifications were from five biological repeats (n = 5). *P < 0.05 versus “aav-Vec” group. Scale bar = 100 μm.

To further substantiate the role of Gαi2 in angiogenesis i*n vivo*, we performed an intravitreal injection of a murine AAV5-TIE1-Gαi2-expressing construct, referred to as “Gαi2-eOE”, in adult mice to induce over-expression of Gαi2 in endothelial cells. Control mice received intravitreal injection of AAV5-TIE1-Vec (“aav-Vec”). Following the injections, we collected and analyzed retinal tissues. Our findings revealed a significant increase in *Gαi2* mRNA (Figure **9A**) and protein (Figure **9B**) expression in the retinal tissues of Gαi2-eOE mice. The expression of Gαi1 and Gαi3 remained unchanged (Figure **9A** and **B**). Furthermore, the expression of NFAT-dependent genes, specifically *Egr3*, *CXCR7*, and *RND1*, exhibited an upregulation in the retinal tissues of Gαi2-eOE mice (Figure **9C**), suggesting the activation of NFAT signaling. Visual examination of retinal vasculature through IB4 staining confirmed a significant increase in angiogenesis in the mouse retina following endothelial over-expression of Gαi2 (Figure **9D**). Gαi2-eOE mice displayed a marked increase in the number of retinal vascular branches and branch points (Figure **9D**). Additionally, vWF and VCAM-1 protein expression was also elevated (Figure **9E**). These findings collectively support that endothelial over-expression of Gαi2 enhances retinal angiogenesis in mice.

### Endothelial Gαi2 silencing ameliorates retinal pathological angiogenesis in diabetic retinopathy mice

**Figure 10:**
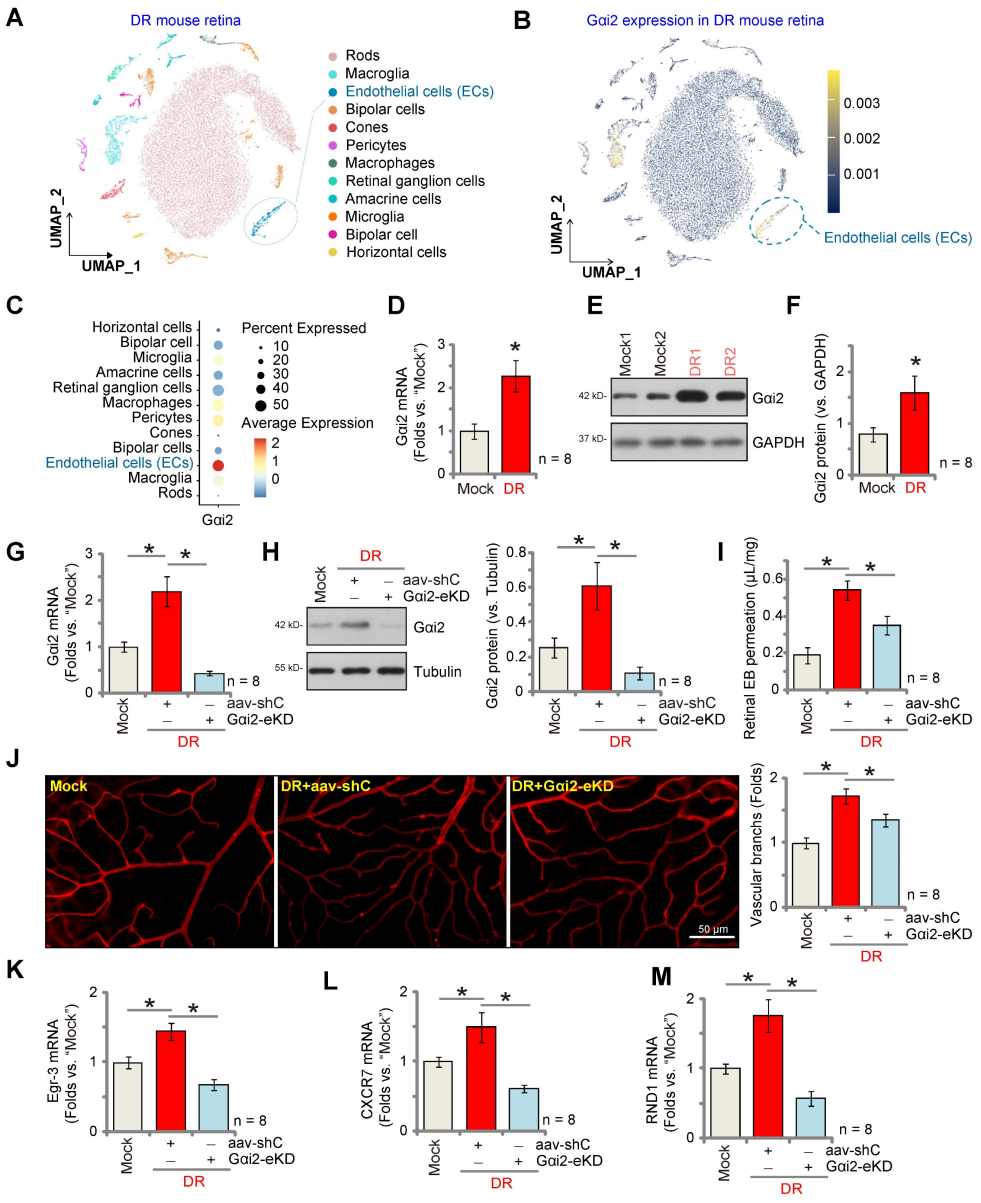
** Endothelial Gαi2 silencing ameliorates retinal pathological angiogenesis in diabetic retinopathy mice.** The publicly accessible scRNA-seq data (GEO: #GSE209872) obtained from STZ-induced diabetic mice through Uniform Manifold Approximation and Projection (UMAP) projections were displayed (**A**). The density plot reveals the expression and spatial distribution of Gαi2 in retinas, with the expression density scale depicted on the right side of the plot (**B**). Analysis based on single-cell data demonstrates the expression profiles of Gαi2 across different retinal cell types in STZ mice (**C**). Ninety days post the final STZ administration, retinal tissues from diabetic retinopathy ("DR") and "Mock" control mice (31.94 ± 1.98 day old, half male half female) were collected for the analysis of *Gαi2* mRNA and protein expression (**D**-**F**). Thirty days after the last STZ treatment, mice received intravitreal injections of murine AAV5-TIE1-Gαi2 shRNA ("Gαi2-eKD", 0.1 μL) or AAV5-containing scramble control shRNA ("aav-shC", 0.1 μL). After an additional 60 days, examination of listed mRNA and protein expression in retinal tissues was conducted (**G**, **H**, **K**-**M**). Alternatively, mice underwent Evans blue (EB) infusion for 2h, and the subsequent EB leakage was quantified (**I**). IB4 staining was conducted to visualize the retinal vasculature (**J**, scale bar = 50 μm). "Mock" refers to mice administered with citrate buffer. Each group consisted of eight mice (n = 8). *P< 0.05 compared to "Mock" (**D**-**F**). *P< 0.05 (**G**-**M**).

Pathological angiogenesis is a key pathology of diabetic retinopathy (DR) 48-53. We next explored the potential role of Gαi2 in the process. We first utilized publicly available single-cell RNA sequencing data (GEO: #GSE209872) obtained from STZ-induced diabetic mice to delineate the cellular localization of Gαi2 within the diabetic retina. Employing reduction in dimensions, clustering techniques, and annotation, the individual cells across the retina were segregated into 12 distinct clusters, which were subsequently visualized using UMAP projections (Figure **10A**). Next, through specifically focusing on Gαi2 expression, our analysis revealed a significant presence of Gαi2 within endothelial cells (Figure **10B** and **C**). This observation underscores the specific localization of Gαi2 in endothelial cells within the diabetic retina in mice.

Next, we assessed Gαi2 expression in mice subjected to streptozotocin (STZ) injections. Retinal tissues from both STZ-injected diabetic retinopathy (DR) mice and control mice were collected 90 days post the final STZ injection. In the DR mouse retinal tissues, there was a significant increase in *Gαi2* mRNA expression (Figure **10D**). Additionally, Gαi2 protein upregulation was observed in retinal tissues of two representative DR mice (Figure **10E**). Combining data from all eight sets of blots revealed a significant upregulation of Gαi2 protein in retinal tissues of STZ-induced DR mice (Figure **10F**).

To investigate the potential involvement of increased Gαi2 expression in the abnormal retinal angiogenesis observed in DR mice, an intravitreal injection of AAV5-TIE1-Gαi2 shRNA was administered into the retinas of DR mice ("Gαi2-eKD") 30 days after the last STZ treatment. Control DR mice received an intravitreal injection of AAV5-TIE1 scramble control shRNA (“aav-shC”). After an additional 60 days, fresh retinal tissues were collected and analyzed. Results demonstrated a reduction in both *Gαi2* mRNA and protein expression in the retinal tissues of Gαi2-eKD DR mice (Figure **10G** and **H**).

When compared to the Mock control mice, there was an increase in retinal vascular leakage measured by Evans blue (EB) staining in AAV-shC DR mice (Figure **10I**). Furthermore, IB4 staining revealed increased vascular complexity (angiogenesis) in the retinas of DR mice (Figure **10J**). Significantly, Gαi2-eKD markedly suppressed the pathological retinal angiogenesis in DR mice (Figure **10I** and **J**), reducing vascular leakage (Figure **10I**) and diminishing vascular complexity (Figure **10J**). Additionally, within the retinal tissue of aav-shC DR mice, there was a significant increase in the expression of NFAT-dependent genes (*Egr3*, *CXCR7*, and *RND1*) (Figure **10K**-**M**), which was abolished following Gαi2-eKD treatment (Figure **10K**-**M**). Collectively, these results provide support that increased expression of Gαi2 played a significant role in the pathological retinal angiogenesis observed in DR mice.

## Discussion

Our recent research underscores the crucial roles of Gαi1 and Gαi3, two other members of Gαi protein family, in angiogenesis 9-13. These proteins are essential for angiogenesis induced by stem cell factor (SCF) via interacting with the SCF-activated receptor c-Kit, initiating Akt-mTOR cascade activation in endothelial cells 10. Silencing, KO (via CRISPR/Cas9), or introducing dominant negative mutations for Gαi1 and Gαi3 significantly impeded SCF-induced Akt-mTOR activation and angiogenesis 10. Gαi1 and Gαi3 also form a complex with Netrin-1-stimulated receptor CD146, promoting angiogenesis *in vitro* and *in vivo* through downstream Akt-mTOR activation 11. Moreover, phosphoenolpyruvate carboxykinase 1 (PCK1) regulates Gαi3 expression and downstream Akt-mTOR activation by enhancing the transcription activity of GATA binding protein 4 (GATA4) 12. Our research also shows the essential roles of Gαi1 and Gαi3 in mediating vascular endothelial growth factor (VEGF)-induced VEGFR2 endocytosis, leading to downstream Akt-mTOR and Erk-MAPK activation, thereby promoting angiogenesis 13. In addition, our findings emphasize the importance of Gαi1 and Gαi3's interaction with the RSPO3 (R-spondin 3)-activated receptor leucine-rich repeat G protein-coupled receptor 4 (LGR4) for downstream Akt-mTOR activation and angiogenesis 9.

In the present study, we demonstrate the pivotal role of Gαi2 in the activation of endothelial cells and the promotion of angiogenesis. Analysis of single-cell RNA sequencing data revealed increased *Gαi2* expression in endothelial cells of OIR mice. Moreover, transcriptome analysis linking *Gαi2* to angiogenesis-related processes/pathways, supported by increased Gαi2 expression in experimental OIR mouse retinal tissues, highlights its role in angiogenesis.

Using various endothelial cell types, including HUVECs, hRMECs, and hCMEC/D3, we employed targeted shRNA silencing and CRISPR/Cas9 KO techniques to suppress Gαi2 expression. Gαi2 deficiency led to a robust reduction in cell proliferation, migration, invasion, and capillary tube formation. Additionally, a notable increase in apoptosis was observed in Gαi2-silenced or -KO endothelial cells. Conversely, ectopic over-expression of Gαi2 displayed pro-angiogenic effects in cultured endothelial cells, enhancing cell proliferation, migration, invasion, and capillary tube formation. Contrarily, the introduction of a dominant negative mutation in Gαi2 effectively inhibited endothelial cell activation. *In vivo* experiments using endothelial-specific Gαi2 shRNA AAV inhibited retinal angiogenesis in mouse retinal tissues. Conversely, endothelial Gαi2 over-expression strengthened retinal angiogenesis in mice. Thus, Gαi2 is essential for endothelial cell activation and angiogenesis.

NFAT is a family of transcription factors central to the regulation of gene expression in immune cells, particularly T lymphocytes 54-56. Upon activation, NFAT is translocated from the cytoplasm to the nucleus, where it orchestrates the transcription of various genes (such as various cytokines) 54-56. NFAT activation is tightly controlled, primarily through signaling pathways including the calcium-calcineurin pathway 54-56. NFAT transcription factors exist in multiple isoforms and are not limited to T cells, as they play roles in B cell activation, mast cell degranulation, neuronal plasticity, carcinogenesis, muscle development and other processes 54-58.

NFAT plays a significant role in endothelial cell activation and angiogenesis. When endothelial cells are exposed to specific stimuli, NFAT promotes the expression of genes essential for endothelial cell activation 39-41, 59. Suehiro *et al.,* have shown that VEGF activates the NFAT cascade in endothelial cells and induces NFATc1 binding of its targeted genes, including *CXCR7* and *RND1*, thereby promoting their expression 40. VEGF activates NFAT and increases it binding with the target gene* Egr-3*, thereby promoting expression of *Egr-3* 41. The latter is required for endothelial cell proliferation, migration, and tube formation 41. Armesilla *et al.,* also showed that VEGF induced NFAT dephosphorylation and nuclear translation, thereby increasing tissue factor (TF) promoter activity and expression 59. CsA, an established NFAT inhibitor, or dominant negative mutation of NFAT inhibits VEGF-induced TF promoter activation in HUVECs 59. Hernández and colleagues reported that NFAT inhibition by CsA suppresses VEGF-induced endothelial cell activation and hinders angiogenesis 39.

In skeletal muscle cells Gαi2 triggers phospholipase C (PLC) activation, leading to the production of inositol trisphosphate (IP3) and subsequent release of intracellular calcium (Ca^2+^) 38. The latter then activates calcineurin, a phosphatase that dephosphorylates NFAT proteins 38. Dephosphorylated NFAT then translocates from the cytoplasm to the nucleus, where it modulates targeted gene expression 38. Our results indicate that Gαi2-driven endothelial cell activation and angiogenesis are primarily mediated by promoting NFAT activation. In HUVECs, Gαi2 silencing, KO or dominant negative mutation results in decreased NFAT-luciferase reporter activity and down-regulation of NFAT targets (*Egr3*, *CXCR7*, and *RND1*), whereas Gαi2 over-expression leads to their upregulation. Notably, the calcineurin-NFAT activator ionomycin rescues NFAT activity, significantly mitigating the inhibitory effects of Gαi2 shRNA on HUVECs proliferation, migration, and tube formation. Conversely, the enhancement in HUVECs proliferation, migration and tube formation induced by Gαi2 over-expression were reversed by the NFAT inhibitor CsA. *In vivo*, down-regulation of NFAT target genes (*Egr3*, *CXCR7*, and *RND1*) in mouse retinal tissues with endothelial knockdown of Gαi2 was detected. Whereas NFAT-dependent genes were up-regulated in mouse retinal tissues with endothelial Gαi2 over-expression. NFAT-dependent genes were also up-regulated in retinal tissues of DR mice. Therefore, Gαi2-mediated endothelial cell activation and angiogenesis are associated with NFAT activation.

DR is a complex condition characterized by two main stages: non-proliferative diabetic retinopathy (NPDR) and proliferative diabetic retinopathy (PDR) 48-50, 60. The transition from NPDR to PDR marks the appearance of abnormal, delicate blood vessels in the retina, prone to leakage and causing issues like retinal edema, hemorrhage, fibrovascular growth, and retinal detachment 48-50, 60-62. This pathological angiogenesis primarily arises from damage to blood vessels due to long-term high blood sugar levels, exacerbated by inflammatory and oxidative stress factors 48-50, 60-62. The interaction between these harmful elements triggers the increase of key agents promoting blood vessel growth, including VEGF, leading to the formation of abnormal blood vessels in the oxygen-deprived retinal environment 52, 53, 60, 63.

Here, examination of single-cell RNA sequencing data and experiments demonstrated significantly increased levels of Gαi2 specifically in retinal endothelial cells of mice with STZ-induced DR. Importantly, targeted silencing of Gαi2 in retinal endothelial cells, achieved through intravitreous administration of AAV5-TIE1-Gαi2 shRNA, effectively mitigated pathological retinal angiogenesis in the DR murine models. Based on these findings, we propose that increased Gαi2 over-expression plays a pivotal role in pathological angiogenesis in DR, emerging as a promising and relevant therapeutic target.

## Supplementary Material

Supplementary figure.
