# Supplementary figures and images for: G protein subunit alpha i2's pivotal role in angiogenesis
Source: Theranostics. 2024 Mar 3;14(5):2190–209. doi: 10.7150/thno.92909 (PMC10945342; doi:10.7150/thno.92909)

Figure S1

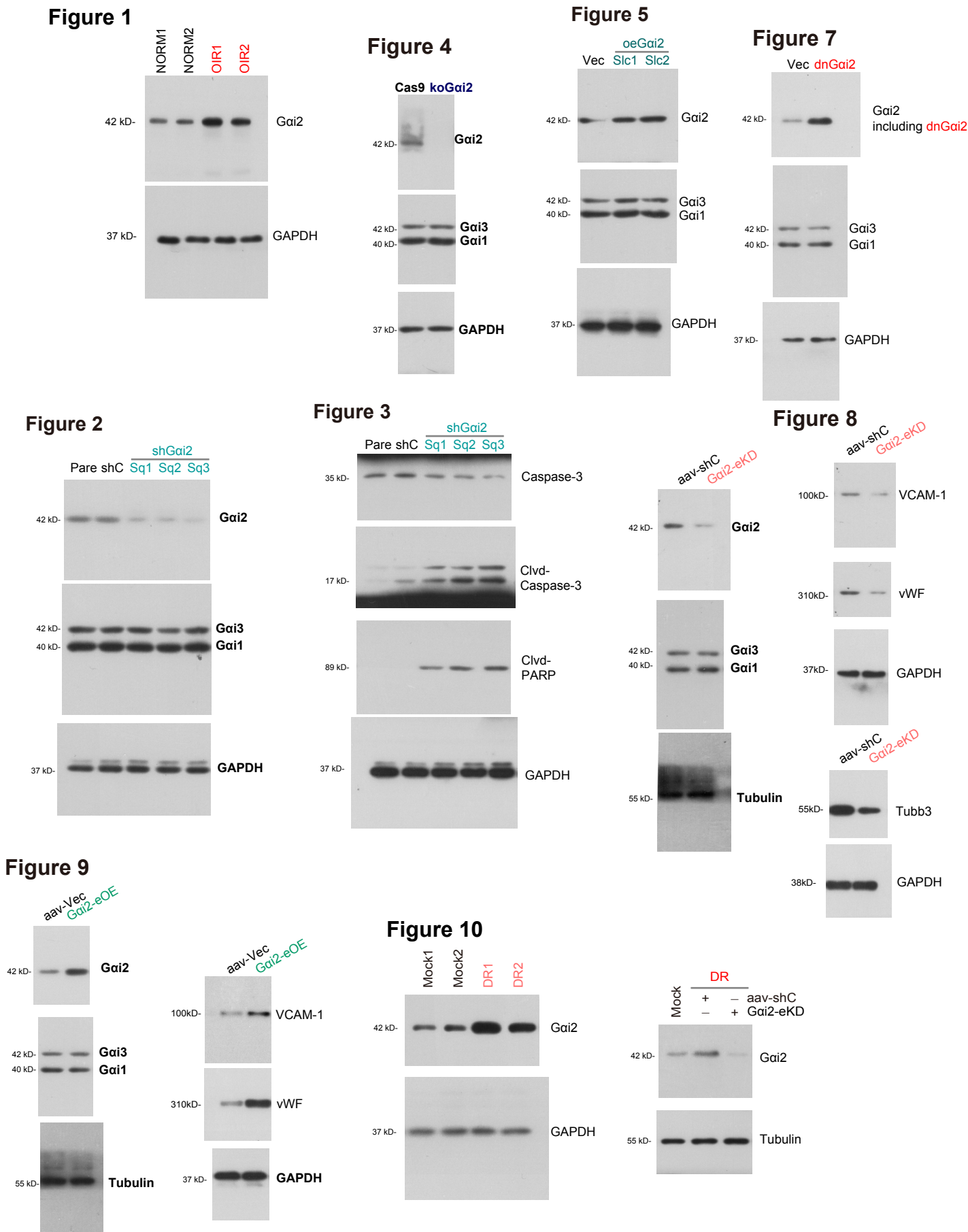

Figure S1. The uncropped blotting images of the study.

Supplement: Supplementary file 1 — Supplementary figure. [file thnov14p2190s1.pdf]
